# Optimal Phosphate Concentration for Growth and Normal Functioning of Marine Anammox Bacteria, *Candidatus* Scalindua sp.

**DOI:** 10.1264/jsme2.ME25042

**Published:** 2025-11-12

**Authors:** Thelwadanage Nadisha Tharangani Kumari Nawarathna, Haruhi Iida, Naoki Fujii, Noriatsu Ozaki, Akiyoshi Ohashi, Jonathan A.C. Roques, Tomonori Kindaichi

**Affiliations:** 1 Department of Civil and Environmental Engineering, Graduate School of Advanced Science and Engineering, Hiroshima University, 1–4–1, Kagamiyama, Higashihiroshima, Hiroshima 739–8527, Japan; 2 Department of Life and Environmental Sciences, Prefectural University of Hiroshima, Nanatsuka 5562, Shobara, Hiroshima 727–0023, Japan; 3 Department of Biological and Environmental Sciences, University of Gothenburg, Box 463, 405 30, Gothenburg, Sweden; 4 Swedish Mariculture Research Center (SWEMARC), University of Gothenburg, Box 463, 405 30, Gothenburg, Sweden; 5 Blue Food Center, University of Gothenburg, Box 463, 405 30, Gothenburg, Sweden

**Keywords:** Anammox, *Candidatus* Scalindua, phosphate, average growth rate, doubling time

## Abstract

The anammox process using marine anammox bacteria is a promising nitrogen removal process for recirculating aquaculture system wastewater. Marine anammox bacteria are typically found in oxygen-deficient zones and coastal areas under low phosphate concentrations. The optimal phosphate concentration for marine anammox bacteria remains unknown because most laboratory studies on these bacteria have been conducted under high phosphate concentrations. Therefore, the present study investigated the long-term effects of varying phosphate concentrations on the marine anammox bacteria, *Candidatus* Scalindua sp., to identify the optimal range of phosphate. Anammox activity and average growth rates were evaluated under seven phosphate concentrations (0, 0.23, 0.46, 0.68, 1.14, 6.15 [control], and 15.48‍ ‍mg P L^–1^) over a period of 70 days. After 50 days of reactor operation, reactor performance under phosphate concentrations ranging from 0.23 to 6.15‍ ‍mg P L^–1^ stabilized at 70% of total nitrogen removal efficiency, indicating the successful establishment of the anammox process. Conversely, anammox reactor performance under conditions without phosphate addition (0‍ ‍mg P L^–1^) and the highest phosphate concentration (15.48‍ ‍mg P L^–1^) did not reach 70% of total nitrogen removal efficiency, indicating a suboptimal phosphate concentration for normal anammox activity. Average growth rates calculated from total biomass samples varied from 0.0006 to 0.0012 h^–1^. These results indicate that *Ca.* Scalindua need to be kept at phosphate concentrations between 0.23 and 6.15‍ ‍mg P L^–1^ for optimal functioning in wastewater treatment ecosystems.

Since its discovery in the early 1990s, the anaerobic ammonium-oxidizing (anammox) process has become a highly active area of research (Ni and Zhang, 2013). This process is contrasted with traditional nitrogen removal processes, such as nitrification and denitrification, in freshwater and marine ecosystems (Lackner *et al.*, 2014). The marine anammox bacteria, *Candidatus* Scalindua are pivotal to the functioning of marine nitrogen cycling, being responsible for 30–50% of nitrogen removal from marine ecosystems (Dalsgaard *et al.*, 2005). These bacteria thrive in oxygen-limited environments, primarily within marine sediments and deep ocean water columns (Ponce-Jahen *et al.*, 2024). Their community structures exhibit ecological variations that are affected by pH, geochemical elements, moisture, and terrestrial location (Liu *et al.*, 2018).

The marine anammox process holds significant promise for wastewater treatment, particularly for saline ammonia-rich effluents, such as municipal and aquacultural wastewater (Tom *et al.*, 2021; Isaka *et al.*, 2022; Ismail *et al.*, 2022; Micolucci *et al.*, 2023; Roques *et al.*, 2024), due to high tolerance to salinity (0.5–3.5%) (Awata *et al.*, 2015; Okabe *et al.*, 2025). Despite their utility, marine anammox bacteria are characterized by slow growth rates, specialized biochemical processes, and sensitivity to environmental fluctuations (van der Star *et al.*, 2007; Ding *et al.*, 2024). However, understanding and optimizing ecological conditions to enhance the anammox process remain challenging.

Phosphate is a vital nutrient for bacterial survival and function (Zheng *et al.*, 2019). It is a key component in energy transfer (*e.g.*, ATP synthesis) and a structural element in DNA and RNA (Nath, 2023). Due to limited phosphate availability in natural environments, bacteria have evolved a number of mechanisms, such as free phosphate uptake, phosphate solubilization, and the utilization of organic phosphate (Alori *et al.*, 2017; Liu *et al.*, 2024). The optimal phosphate concentration may vary between freshwater and marine anammox bacteria due to differences in their ecological environments. The marine anammox, *Ca.* Scalindua have adapted to environments with lower phosphate concentrations due to the oligotrophic nature of oceanic water, where phosphate is often a limiting nutrient (Guilbaud *et al.*, 2020; Planavsky *et al.*, 2023). Nevertheless, the activity of *Ca.* Scalindua remains significant even at extremely low phosphate concentrations (Okabe *et al.*, 2024). However, sub-optimal phosphate availability may negatively affect the growth and function of *Ca.* Scalindua (Zhang *et al.*, 2017b). A minimal phosphate concentration is often associated with reductions in both ribosome production and growth rates, while an excessive phosphate concentration may inhibit bacterial activity (Juhna *et al.*, 2007; Yao *et al.*, 2016). Moreover, long-term studies suggested no adverse effects on reactor performance at phosphate concentrations up to 500‍ ‍mg P L^–1^ in freshwater anammox bacteria (Zhang *et al.*, 2016). However, anammox activity was found to decrease by 37% at phosphate levels of 55.7‍ ‍mg PO_4_^3–^ L^–1^ (17.6‍ ‍mg P L^–1^) and by 80% at 111.5‍ ‍mg PO_4_^3–^ L^–1^ (35.4‍ ‍mg‍ ‍P‍ ‍L^–1^) (Pynaert *et al.*, 2003). Furthermore, the minimum phosphate concentration (MPC) required for the activity of freshwater anammox bacteria corresponds to an optimal MPC-to-nitrogen ratio of 0.0005 (Tomisaki *et al.*, 2023). Limited information is currently available on optimal phosphate concentrations for marine anammox bacteria. *Ca.*‍ ‍Scalindua previously exhibited enhanced activity at‍ ‍phosphate concentrations of 5 to 30‍ ‍mg PO_4_^3–^ L^–1^ (1.6–‍9.5‍ ‍mg‍ ‍P‍ ‍L^–1^) (Si *et al.*, 2021). To the best of our knowledge, variations in growth rates, doubling times, and the MPC levels of *Ca.* Scalindua at different phosphate concentrations have not yet been exami­ned.

Therefore, the present study investigated the long-term effects of various phosphate concentrations on the anammox activity and average growth rate of *Ca.* Scalindua. To achieve this, we employed long-term, continuously fed up-flow column reactors because continuous column reactors have an advantage in assessing the growth potential of slow-growing *Ca.* Scalindua. To monitor and quantify the 16S rRNA gene copies of *Ca.* Scalindua and evaluate their average growth rates, a qPCR anal­ysis was conducted. Reactor biomass samples were collected at the end of reactor operation for a period of 70 days, while effluent samples were collected at 10-day intervals. Average growth rates and doubling times were calculated based on the 16S rRNA gene copies of *Ca.* Scalindua and were compared using a microbial community anal­ysis.

## Materials and Methods

### Reactor design and operation conditions

Prior to the continuous experiment, we conducted an abiotic experiment to confirm optimal phosphate and bicarbonate concentrations in the reactor because high phosphate concentrations may induce chemical precipitation. The abiotic experiment was performed with three 300-mL glass up-flow column reactors (Run P1–Run P3, KF-30; AS ONE) and three different phosphate and bicarbonate concentrations as shown in Table S1, based on modified medium compositions from previous studies (Awata *et al.*, 2015). The non-woven fabric sheet (Kindaichi *et al.*, 2011a) was fixed in the column reactor without the introduction of an anammox biomass. The reactors were operated at 28°C with a continuous flow rate of 3‍ ‍mL‍ ‍min^–1^, maintaining a pH of 7.5±0.2 by adding sulfuric acid (van de Graaf *et al.*, 1996). The hydraulic retention time (HRT) was kept at 1.5 h. Phosphate precipitation within the reactor was evaluated as the difference between influent and effluent phosphate concentrations. Differences between influent and effluent phosphate concentrations decreased with increases in KHCO_3_ concentrations (Fig. S1A). Phosphate precipitation was negligible at 1,500‍ ‍mg L^–1^ of KHCO_3_ (Fig. S1B). Therefore, we conducted further experiments with 1,500‍ ‍mg L^–1^ of KHCO_3_ to minimize chemical precipitation in the reactor, even though the concentration of KHCO_3_ was three-fold higher than that in a previous study (Kindaichi *et al.*, 2011a). We also assumed that phosphate precipitation enhanced the granulation of *Ca.* Scalindua, thereby reducing biomass washout and increasing biomass retention. While the present study provides novel insights, further anal­yses are needed to fully elucidate the underlying mechanisms. The synthetic wastewater for the abiotic experiment was flushed with nitrogen gas for at least 1‍ ‍h to obtain a dissolved oxygen concentration <0.5‍ ‍mg L^–1^ (van de Graaf *et al.*, 1996; Roques *et al.*, 2024).

The continuous experiment was performed with seven 300-mL glass up-flow column reactors (Run 1–Run 7, KF-30; AS ONE) operated under a plug-flow configuration with a short HRT. Synthetic wastewater containing 35‍ ‍g‍ ‍L^–1^ artificial sea salt (SEALIFE; Marinetech), 31.8‍ ‍mg N L^–1^ (NH_4_)_2_SO_4_, and 30.4‍ ‍mg N L^–1^ NaNO_2_ was used in this study (Table 1). The non-woven fabric sheet (Kindaichi *et al.*, 2011b) was fixed inside the reactor as a biomass carrier. Each reactor was inoculated with 1‍ ‍g wet weight (approximately 0.16‍ ‍g dry weight) of a marine anammox biomass, collected from a parent reactor and a homogenized sample using a Dounce Tissue Grinder (Wheaton), in which the phosphate concentration was maintained at 6.15‍ ‍mg P L^–1^ for more than 15 years (Kindaichi *et al.*, 2011a). The seven reactors were operated at 28°C for 70 days with a continuous flow rate of 3‍ ‍mL‍ ‍min^–1^, maintaining a pH of 7.5±0.2 by adding sulfuric acid. The HRT was kept at 1.5 h. Salt solutions (MgSO_4_ and CaCl_2_) and trace element solutions (TE I and TE II) were used as previously described by van de Graaf *et al.* (1996). Synthetic wastewater for the continuous experiment was flushed with nitrogen gas for at least 1‍ ‍h to reduce the dissolved oxygen concentration to lower than 0.5‍ ‍mg L^–1^ (van de Graaf *et al.*, 1996; Roques *et al.*, 2024).

### Analytical methods

The concentration of NH_4_^+^ was measured using Nessler’s method (Hach method 8038) with a UV-visible spectrophotometer (DR 2800; Hach-Lange) (Kambara *et al.*, 2022). To assess NO_2_^–^ and NO_3_^–^, samples were prepared by filtering through a cellulose acetate membrane with a pore size of 0.2‍ ‍μm (Advantec). We then measured NO_2_^–^ and NO_3_^–^ concentrations using ion chromatography (HPLC 20A; Shimadzu) (Roques *et al.*, 2024). The phosphate concentration was assessed using a Hach-Lange water quality analyzer equipped with a UV-Visible spectrophotometer following Hach method 8048 with PhosVer^®^3 phosphate reagent powder pillows (DR 2800; Hach-Lange, Nurmiyanto *et al.*, 2017).

### Effluent and reactor biomass sample collection and DNA extraction

Every 10 days, 1,000‍ ‍mL of an effluent sample was collected from each reactor (seven effluent samples per reactor) and filtered through a polytetrafluorethylene (PTFE) membrane filter with a diameter of 47‍ ‍mm and pore size of 0.5‍ ‍μm (Advantec). The filter was dried in a freeze dryer (DC401; Yamato Scientific). The weight of the dried biomass from the filter was measured, and dry biomass samples were used for DNA extraction. At the end of the reactor operation (after 70 days), the non-woven fabric sheet with the attached biomass was collected from each reactor, the fabric sheet was dried (DC401; Yamato Scientific) and the reactor biomass sample (*i.e.*, total inside biomass sample) from each reactor was collected from the non-woven fabric sheet. The total bacteria DNA of each effluent, reactor biomass sample, and initial sample (*i.e.*, the inoculum) was extracted using a QIAGEN Genomic-tip 20/G kit (QIAGEN GmbH) according to the manufacturer’s instructions. DNA concentrations were measured using a Qubit 2.0 fluorometer (Life Technologies).

### Quantification of marine anammox bacteria by a qPCR anal­ysis

qPCR assays were performed using the TaqMan method to amplify the 16S rRNA gene using the Applied Biosystem Step One Real-Time PCR system (Life Technologies). Each 20-μL reaction mixture contained 2‍ ‍μL of extracted template DNA, 10‍ ‍μL of the‍ ‍THUNDERBIRD Probe qPCR Mix (TOYOBO), 0.4‍ ‍μL of 50×‍ ‍ROX reference dye (TOYOBO), 0.5‍ ‍μL of each forward (SCJ447F) and reverse (SCJ629R) primer (Zhang *et al.*, 2017a), 0.5‍ ‍μL of the TaqMan probe (SCJ512P) (Zhang *et al.*, 2017a), and 6.1‍ ‍μL of PCR-grade water (Nacalai Tesque). qPCR was conducted on a 48-well reaction plate under the following conditions: holding stage at 95°C for 60‍ ‍s, followed by 40 cycles of the cycling stage at 95°C for 15‍ ‍s and 60°C for 60 s. All qPCR runs were conducted in triplicate with non-template negative controls. Standard curves were obtained with a plasmid containing the 16S rRNA partial sequence *Ca.* Scalindua covering the primer region (Eurofins Genomics) as shown in Fig. S2.

### Microbial community anal­ysis

PCR amplification of the bacterial 16S rRNA gene was performed with a primer set for the amplification of the V3-V4 region as follows: 341F (5′-CCTACGGGNGGCWGCAG-3′) and 805R (5′-GGACTACHVGGGTATCTAATCC-3′). The details of PCR amplification were as previously described (Shoiful *et al.*, 2020). According to the manufacturer’s instructions, PCR products were purified using the Agencourt AMPure XP system (Beckman Coulter). Purified DNA was sequenced using a MiSeq platform with a MiSeq reagent kit (v.3; Illumina). The sequences obtained were trimmed and assembled as previously described (Awata *et al.*, 2021; Fujii *et al.*, 2022). Sequence data were analyzed using the QIIME 2 Core 2023.2 distribution (Bolyen *et al.*, 2019). Amplicon sequence variants (ASV) were assigned to the SILVA 138.1 database (Quast *et al.*, 2012).

### Estimations of the average growth rate (μ) and doubling time (T_d_)

The average growth rate (μ) was calculated using the slope of biomass concentrations between selected exponential growth phases, as shown in Eq. (1)

$$\mu =(lnx_{2}-lnx_{1\;})/(t_{2}-t_{1})$$

 (1)

where μ is the specific growth rate (h^–1^), x_1_ and x_2_ are cell concentrations, *i.e.*, 16S rRNA gene copies of *Ca.* Scalindua per 1,000‍ ‍mL of the effluent (copies L effluent^–1^) at operation times t_1_ and t_2_ (h). The doubling time (T_d_) was calculated using Eq. (2)

$$T_{d}=\frac{ln2}{\mu }$$

 (2)

To convert gene copy numbers to cell numbers, we used a 1:1 ratio of 16S rRNA gene copies to *Ca.* Scalindua cells based on a previous metagenomic anal­ysis (Nawarathna *et al.*, 2025).

## Results and Discussion

### Long-term nitrogen removal performance under various phosphate concentrations

**Fig. 1.**
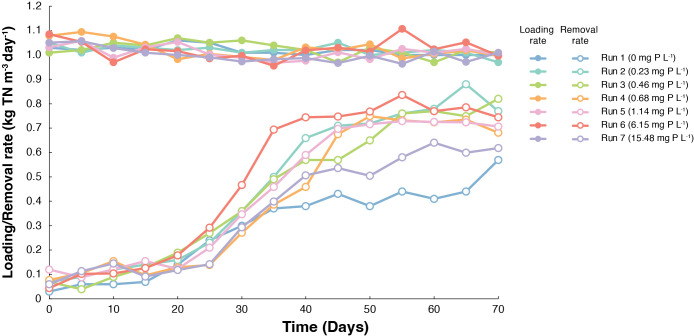
Nitrogen removal performance of up-flow column reactors under various phosphate concentrations.

The nitrogen loading rates of seven reactors were maintained at 1.0±0.03‍ ‍kg TN m^–3^ day^–1^, while nitrogen removal rates varied with different phosphate concentrations (Fig. 1). After 10–20 days, nitrogen removal rates increased in all reactors. This result suggests that different phosphate concentrations did not enhance anammox performance during the initial growth phase. Run 6 (6.15‍ ‍mg P L^–1^) was used as the control reactor because the parent reactor (*i.e.*, the inoculum in this study) had been operated at this phosphate concentration for more than 15 years (Kindaichi *et al.*, 2011b). In Run 2 (0.23‍ ‍mg P L^–1^) to Run 6 (6.15‍ ‍mg P L^–1^), total nitrogen removal (TN) rates successfully increased from 0.65 to 0.77‍ ‍kg TN m^–3^ day^–1^ with high removal efficiencies of >57% for NH_4_^+^ (Fig. S3A) and >85% for NO_2_^–^ (Fig. S3B) at the end of 50 days. However, Run 1 (no addition of phosphate) resulted in a lower TN removal rate of 0.38‍ ‍kg‍ ‍TN‍ ‍m^–3^‍ ‍day^–1^ with low removal efficiencies of 28% for NH_4_^+^ and 53% for NO_2_^–^ after 50 days, indicating a deficiency of phosphate for anammox activity. Furthermore, Run 7 (15.48‍ ‍mg P L^–1^) achieved a TN removal rate of 0.50‍ ‍kg TN m^–3^ day^–1^ with a 50% NH_4_^+^ removal efficiency and 70% NO_2_^–^ removal efficiency after 50 days, indicating moderate anammox performance. The anammox stoichiometric ratios of Run 2–Run 7 shown in Fig. S3C were close to the values for 70 days of operation (1.32 for ΔNO_2_^–^/ΔNH_4_^+^ and 0.26 for ΔNO_3_^–^/ΔNH_4_^+^). On the other hand, the anammox stoichiometric ratios of Run 1 were not close to 1.32. This result also indicated a deficiency of phosphate for anammox activity.

As previously mentioned, Si *et al.* (2021) proposed that marine anammox bacteria thrive within phosphate concentrations of 5 to 30‍ ‍mg PO_4_^3–^ L^–1^ (1.6 to 9.5‍ ‍mg P L^–1^). The present study extends this finding by demonstrating significant activity across a broader range, from 0.7‍ ‍mg PO_4_^3–^ L^–1^ (0.23‍ ‍mg P L^–1^) to 47‍ ‍mg PO_4_^3–^ L^–1^ (15.48‍ ‍mg P L^–1^). Marine anammox bacteria exhibit greater stability across varying phosphate concentrations than freshwater anammox bacteria because many marine bacterial communities often display functional stability under varying phosphate concentrations due to physiological adaptations, such as utilizing organic phosphate sources (Adams *et al.*, 2022). Additionally, bacteria may regulate their metabolism to cope with phosphate fluctuations by modifying their membrane lipid composition, replacing phospholipids with sulfolipids or glycolipids when phosphate is scarce. Moreover, coexisting bacteria may dominate under high and low phosphate conditions, ensuring the ecosystem (Thingstad *et al.*, 2005; Sebastian and Ammerman, 2009; Van Mooy *et al.*, 2009; Karl, 2014). Furthermore, this enhanced resilience may be due to the formation of biofilms by marine anammox bacteria, which provides greater tolerance to phosphate stress than granular or floc-associated freshwater anammox bacteria (Yang *et al.*, 2019).

### Quantification of *Ca.* Scalindua by a qPCR anal­ysis and microbial community structure

**Fig. 2.**
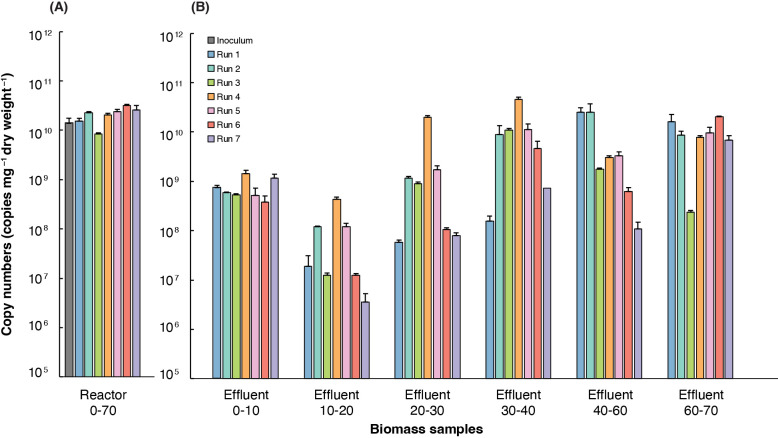
Average copy numbers of the 16S rRNA gene of *Candidatus* Scalindua in reactor biomass (A) and effluent biomass (B) samples. Reactor biomass samples were collected at inoculation and after 70 days (0–70). Effluent samples represent a cumulative copy number for 10 days calculated from 1 L of effluent corrected at 10- or 20-day intervals. For example, Effluent 10–20 refers to the cumulative copy number for 10 days calculated from 1 L of effluent on day 20. Error bars represent the standard deviations of triplicate measurements.

Reactor biomass samples (*i.e.*, total inside biomass samples) in the reactors after 70 days consistently showed the highest 16S rRNA gene copy numbers (10^10^–10^11^) (Fig. 2A). This result indicates effective microbial retention and growth inside the reactor. Furthermore, the biomass samples of all runs, except Run 3, had higher 16S rRNA gene copy numbers than the initial sample. This result demonstrates effective microbial colonization and growth during the experiment.

In the effluent biomass samples across all runs, the copy numbers of *Ca.* Scalindua 16S rRNA slightly increased during the exponential growth phase until 40 days in the Run 2–5 phase (Fig. 2B), but stabilized in the later stages, as observed in Run 4. Copy numbers in effluent samples were lower during 10–20 days than during 0–10 days, which may have been due to the washout of the biomass that was not attached to the fabric sheet during the early stage (0–10 days after the inoculation).

In total effluent biomass samples, we assumed that Runs 2–7 reached the exponential growth phase within 20–30 days (Fig. S4). An interesting result showing exponential growth between 40 and 60 days in Run 1 was obtained, which indicated a delayed response and adaptation with unexpected phosphate zero availability for growth. However, the overall time required to reach the exponential growth phase was long (more than 20 days) because *Ca.* Scalindua is known to have a slow average growth rate and low cell yield (Jin *et al.*, 2012).

**Fig. 3.**
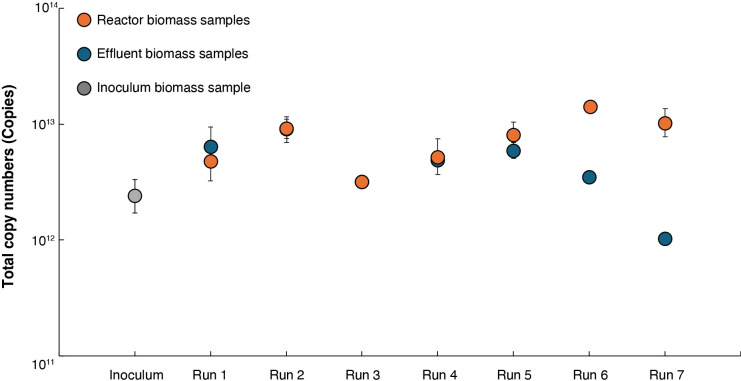
Total copy numbers of the 16S rRNA gene of *Candidatus* Scalindua in the reactor biomass and effluent biomass samples after 70 days of reactor operation. Errors were calculated based on the law of error propagation.

The total copy numbers of the 16S rRNA genes of *Ca.* Scalindua were calculated based on reactor biomass samples obtained after 70 days and effluent biomass samples collected in 10-day intervals. To compare the amount of biomass between reactor and effluent samples, 10-day interval effluent samples were converted to 70-day cumulative values (Fig. 3) with detailed calculations provided in the supplemental method. These results showed that the total copy numbers in Runs 1 to 5 in the reactor biomass and the effluent biomass were similar. The ratios of the total copy numbers in the reactor biomass to the total copy numbers in effluent samples ranged from 1.1 to 1.5, which indicated that 50% of *Ca.* Scalindua cells were washed out from the reactor and were not negligible. In Runs 6 and 7, the total copy numbers in the reactor biomass were higher than those in the effluent samples. The ratios were 4.0 in Run 6 and 10.0 in Run 7. Higher ratios may be attributed to higher phosphate concentrations. High phosphate concentrations often contribute to the induction of genes related to cellular growth and metabolic functions. For example, in *Escherichia coli*, the Pho regulon is controlled by a two-component system (PhoR/PhoB) (Wanner, 1993). The gene abundance of *Ca.* Scalindua increased in some reactors even in the absence of external phosphorus supplementation. Therefore, *Ca.* Scalindua may utilize internal phosphorus sources or rely on recycling mechanisms (cell lysis, extracellular DNA, or organic P compounds) to sustain growth under P-limited conditions (Pinchuk *et al.*, 2008; Mine *et al.*, 2021).

**Fig. 4.**
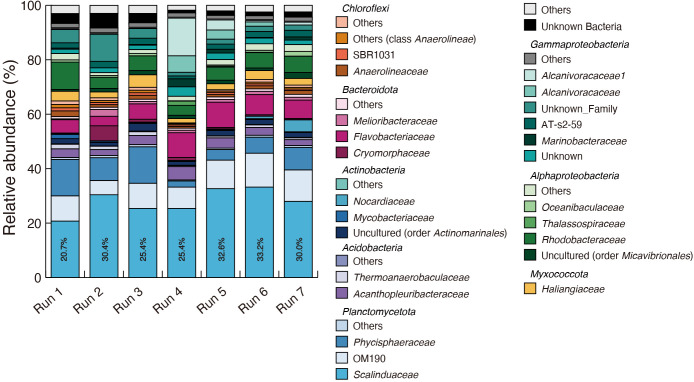
Microbial community compositions of reactor biomass samples after 70 days of reactor operation. Percentages represent the relative abundance of *Candidatus* Scalindua.

A microbial community anal­ysis using reactor biomass samples showed that *Ca.* Scalindua was the dominant bacterial group, ranging from 20.7–33.2% of total bacteria (Fig. 4). In previous studies involving the same *Ca.* Scalindua species, the microbial community accounted for 28.7% after 78 days and 22.7% after 38 days in synthetic marine wastewater, and 16.7% on day 280 in recirculating aquaculture system wastewater (Roques *et al.*, 2021, 2024; Micolucci *et al.*, 2023). The profiles of the relative abundance of *Ca.* Scalindua matched well with total copy numbers in reactor biomass samples, as shown in Fig. 3. The lower abundance of *Ca.* Scalindua in Run 1 may be related to a deficiency of phosphate, with lower removal efficiency. Other bacterial communities coexisting with *Ca.* Scalindua were similar to those observed in previous studies (Roques *et al.*, 2021, 2024; Micolucci *et al.*, 2023, Nawarathna *et al.*, 2025).

### Average growth rate (μ) and doubling time (T_d_)

Average growth rates and doubling times calculated from the total biomass across the seven reactors are summarized in Table 2. Average growth rates varied from 0.0006 to 0.0012 h^–1^, corresponding to doubling times ranging from 23.7 to 48.7 days. Runs 2, 5, and 6 achieved higher growth rates and shorter doubling times, suggesting more favorable operational or environmental conditions for biomass productivity. Run 3 showed lower copy numbers in the inside biomass and effluent biomass samples (Fig. 3) with a lower average growth rate and high doubling time, which indicated suboptimal environmental conditions. These results show that even within the same reactor system, small variations in operational parameters may lead to marked differences in growth kinetics, underscoring the importance of optimizing conditions to enhance marine anammox enrichment. Previous studies reported a maximum growth rate of 0.002 h^–1^ for *Ca.* Scalindua in a membrane bioreactor at 28°C (Awata *et al.*, 2013) and a maximum growth rate of 0.007 h^–1^ at 22°C (Zhang *et al.*, 2017a). However, the overall growth rate in our reactors was low, as indicated by the average growth rate over the 70-day operational period. Fig. S4 shows the profile of 16S rRNA gene copy numbers of *Ca.* Scalindua in the effluent samples, which closely followed reactor performance (Fig. 1), with copy numbers increasing over time. Runs 3–7 reached a saturation state after 70 days of operation, whereas Runs 1 and 2, which had lower phosphate concentrations, were still in the growth phase.

The observed rapid average growth rate of *Ca.* Scalindua under phosphate-limited conditions suggests that a unique ecological niche was created within the reactor environment, allowing the marine anammox bacteria to adapt. The consistent operation without external phosphate addition, along with stable anammox activity, implies that *Ca.* Scalindua possess physiological or metabolic traits that enable efficient phosphorus uptake or recycling. This highlights its ecological competitiveness in low-phosphate saline environments and underscores the potential for practical applications in engineered systems treating saline wastewater where phosphorus levels are typically low. A more detailed understanding of niche adaptation mechanisms may inform reactor design and operational strategies to enhance biomass retention and anammox efficiency.

## Conclusions

In the present study, the growth potential and anammox activity of *Ca.* Scalindua were investigated using up-flow column reactors under seven varying phosphate concentrations. *Ca.* Scalindua exhibited anammox activity and growth under phosphate concentrations ranging from 0.23 (Run 2) to 6.15 (Run 6) mg P L^–1^. The results obtained herein provide valuable insights into the adaptability of *Ca.* Scalindua to phosphate-limited environments, and offer practical guidance for effective bioreactor operation, such as the treatment of salinity wastewater with low phosphate concentrations, operating efficiently without the need for an external phosphate supply. Future research is needed to examine the mechanisms operating under low and high phosphate concentrations with a functional gene expression anal­ysis. Moreover, an assessment of the minimum phosphate concentration required for marine anammox bacteria is essential for their practical application to the treatment of salinity wastewater containing a low phosphate concentration without an external phosphate supply.

### Data availability

Amplicon sequence data are available in the DDBJ database under the accession number PRJDB35461.

## Citation

Nawarathna, T. N. T. K., Iida, H., Fujii, N., Ozaki, N., Ohashi, A., Roques, J. A. C., and Kindaichi, T. (2025) Optimal Phosphate Concentration for Growth and Normal Functioning of Marine Anammox Bacteria, *Candidatus* Scalindua sp.. *Microbes Environ ***40**: ME25042.

https://doi.org/10.1264/jsme2.ME25042

## Supplementary Material

Supplementary Material

## Figures and Tables

**Table 1.** Composition of synthetic wastewater under different phosphate concentrations.

Compounds	Unit	Run 1	Run 2	Run 3	Run 4	Run 5	Run 6	Run 7
SEALIFE	g L^–1^	35	35	35	35	35	35	35
(NH_4_)_2_SO_4_	mg N L^–1^	31.8	31.8	31.8	31.8	31.8	31.8	31.8
NaNO_2_	mg N L^–1^	30.4	30.4	30.4	30.4	30.4	30.4	30.4
KHCO_3_	mg L^–1^	1,500	1,500	1,500	1,500	1,500	1,500	1,500
MgSO_4_·7H_2_O	mg L^–1^	300	300	300	300	300	300	300
CaCl_2_·2H_2_O	mg L^–1^	180	180	180	180	180	180	180
KH_2_PO_4_	mg P L^–1^	0	0.23	0.46	0.68	1.14	6.15	15.48
TE I	mL L^–1^	1	1	1	1	1	1	1
TE II	mL L^–1^	1	1	1	1	1	1	1

**Table 2.** Estimated average growth rate and doubling time for various reactor runs based on reactor biomass and effluent biomass samples after 70 days of operation. Errors are calculated based on the law of error propagation.

Reactor	Average growth rate (h^–1^)	Doubling time (day)
Run 1	0.0009±0.0002	31.2
Run 2	0.0012±0.0002	23.7
Run 3	0.0006±0.0002	48.7
Run 4	0.0009±0.0002	32.7
Run 5	0.0011±0.0002	27.1
Run 6	0.0012±0.0002	24.1
Run 7	0.0009±0.0002	31.0

## References

[B1] Adams, J.C., Steffen, R., Chou, C., Duhamel, S., and Diaz, J.M. (2022) Dissolved organic phosphorus utilization by the marine bacterium *Ruegeria pomeroyi* DSS-3 reveals chain length‐dependent polyphosphate degradation. Environ Microbiol 24: 2259–2269. 35102659 10.1111/1462-2920.15877 PMC9303572

[B2] Alori, E.T., Glick, B.R., and Babalola, O.O. (2017) Microbial phosphorus solubilization and its potential for use in sustainable agriculture. Front Microbiol 8: 971. 28626450 10.3389/fmicb.2017.00971 PMC5454063

[B3] Awata, T., Oshiki, M., Kindaichi, T., Ozaki, N., Ohashi, A., and Okabe, S. (2013) Physiological characterization of an anaerobic ammonium-oxidizing bacterium belonging to the “Candidatus Scalindua” group. Appl Environ Microbiol 79: 4145–4148. 23584767 10.1128/AEM.00056-13 PMC3697556

[B4] Awata, T., Kindaichi, T., Ozaki, N., and Ohashi, A. (2015) Biomass yield efficiency of the Marine Anammox Bacterium, “Candidatus Scalindua sp.,” is affected by salinity. Microbes Environ 30: 86–91. 25740428 10.1264/jsme2.ME14088 PMC4356468

[B5] Awata, T., Goto, Y., Kuratsuka, H., Aoi, Y., Ozaki, N., Ohashi, A., and Kindaichi, T. (2021) Reactor performance and microbial community structure of single-stage partial nitritation anammox membrane bioreactors inoculated with Brocadia and Scalindua enrichment cultures. Biochem Eng J 170: 107991.

[B6] Bolyen, E., Rideout, J.R., Dillon, M.R., Bokulich, N.A., Abnet, C.C., Al-Ghalith, G.A., et al. (2019) Reproducible, interactive, scalable and extensible microbiome data science using QIIME 2. Nat Biotechnol 37: 852–857. 31341288 10.1038/s41587-019-0209-9 PMC7015180

[B7] Dalsgaard, T., Thamdrup, B., and Canfield, D.E. (2005) Anaerobic ammonium oxidation (anammox) in the marine environment. Res Microbiol 156: 457–464. 15862442 10.1016/j.resmic.2005.01.011

[B8] Ding, X., Li, Y., Zhao, J., Miao, Y., Zhao, D., Li, Y., et al. (2024) Self-enriching anammox bacteria and in situ establishing anammox process in traditional wastewater treatment system. J Water Process Eng 68: 106503.

[B9] Fujii, N., Kuroda, K., Narihiro, T., Aoi, Y., Ozaki, N., Ohashi, A., and Kindaichi, T. (2022) Metabolic potential of the superphylum *Patescibacteria* reconstructed from activated sludge samples from a municipal wastewater treatment plant. Microbes Environ 37: ME22012. 35768268 10.1264/jsme2.ME22012 PMC9530719

[B10] Guilbaud, R., Poulton, S.W., Thompson, J., Husband, K.F., Zhu, M., Zhou, Y., et al. (2020) Phosphorus-limited conditions in the early Neoproterozoic ocean maintained low levels of atmospheric oxygen. Nat Geosci 13: 296–301.

[B11] Isaka, K., Sugawara, D., Yamazaki, H., Kimura, Y., Osaka, T., and Tsuneda, S. (2022) Long-term limitation effects of Se (VI), Zn (II), and Ni (II) on start-up of the anammox process using gel carrier. Front Bioeng Biotechnol 10: 851617. 35309992 10.3389/fbioe.2022.851617 PMC8931481

[B12] Ismail, I.N., Taufik, M., Umor, N.A., Norulhuda, M.R., Zulkarnaini, Z., and Ismail, S. (2022) Anammox process for aquaculture wastewater treatment: operational condition, mechanism, and future prospective. Water Sci Technol 86: 3093–3112. 36579872 10.2166/wst.2022.403

[B13] Jin, R.-C., Yang, G.-F., Yu, J.-J., and Zheng, P. (2012) The inhibition of the anammox process: A review. Chem Eng J 197: 67–79.

[B14] Juhna, T., Birzniece, D., and Rubulis, J. (2007) Effect of phosphorus on survival of *Escherichia coli* in drinking water biofilms. Appl Environ Microbiol 73: 3755–3758. 17416695 10.1128/AEM.00313-07 PMC1932671

[B15] Kambara, H., Shinno, T., Matsuura, N., Matsushita, S., Aoi, Y., Kindaichi, T., et al. (2022) Environmental factors affecting the community of methane-oxidizing bacteria. Microbes Environ 37: ME21074. 35342121 10.1264/jsme2.ME21074 PMC8958294

[B16] Karl, D.M. (2014) Microbially mediated transformations of phosphorus in the sea: new views of an old cycle. Ann Rev Mar Sci 6: 279–337. 10.1146/annurev-marine-010213-135046 24405427

[B17] Kindaichi, T., Awata, T., Suzuki, Y., Tanabe, K., Hatamoto, M., Ozaki, N., and Ohashi, A. (2011a) Enrichment using an up-flow column reactor and community structure of marine anammox bacteria from coastal sediment. Microbes Environ 26: 67–73. 21487205 10.1264/jsme2.me10158

[B18] Kindaichi, T., Awata, T., Tanabe, K., Ozaki, N., and Ohashi, A. (2011b) Enrichment of marine anammox bacteria in Hiroshima Bay sediments. Water Sci Technol 63: 964–969. 21411947 10.2166/wst.2011.277

[B19] Lackner, S., Gilbert, E.M., Vlaeminck, S.E., Joss, A., Horn, H., and van Loosdrecht, M.C.M. (2014) Full-scale partial nitritation/anammox experiences—An application survey. Water Res 55: 292–303. 24631878 10.1016/j.watres.2014.02.032

[B20] Liu, J., Xu, W., Zhang, Q., Liao, W., Li, L., Chen, S., et al. (2024) OsPHR2-mediated recruitment of Pseudomonadaceae enhances rice phosphorus uptake. Plant Commun 5: 100930. 38685708 10.1016/j.xplc.2024.100930 PMC11369732

[B21] Liu, K., Ding, X., Tang, X., Wang, J., Li, W., Yan, Q., and Liu, Z. (2018) Macro and microelements drive diversity and composition of prokaryotic and fungal communities in hypersaline sediments and saline–alkaline soils. Front Microbiol 9: 352. 29535703 10.3389/fmicb.2018.00352 PMC5835090

[B22] Micolucci, F., Roques, J.A.C., Ziccardi, G.S., Fujii, N., Sundell, K., and Kindaichi, T. (2023) Candidatus Scalindua, a biological solution to treat saline recirculating aquaculture system wastewater. Processes 11: 690.

[B23] Mine, A.H., Coleman, M.L., and Colman, A.S. (2021) Phosphorus release and regeneration following laboratory lysis of bacterial cells. Front Microbiol 12: 641700. 33897649 10.3389/fmicb.2021.641700 PMC8060472

[B24] Nath, S. (2023) Phosphorus chemistry at the roots of bioenergetics: ligand permutation as the molecular basis of the mechanism of ATP synthesis/hydrolysis by F_O_F_1_-ATP synthase. Molecules 28: 7486. 38005208 10.3390/molecules28227486 PMC10673332

[B25] Nawarathna, T.N.T.K., Fujii, N., Yamamoto, K., Kuroda, K., Narihiro, T., Ozaki, N., et al. (2025) Metagenomic insights into *Candidatus* Scalindua in a long-term cultivated marine anammox consortia: the important role of tetrahydrofolate-mediated carbon fixation. Microbes Environ 40: ME25007. 40533170 10.1264/jsme2.ME25007 PMC12213060

[B26] Ni, S.Q., and Zhang, J. (2013) Anaerobic ammonium oxidation: from laboratory to full-scale application. BioMed Res Int 1: 10. 10.1155/2013/469360 PMC3730388 23956985

[B27] Nurmiyanto, A., Kodera, H., Kindaichi, T., Ozaki, N., Aoi, Y., and Ohashi, A. (2017) Dominant Candidatus; Accumulibacter phosphatis enriched in response to phosphate concentrations in EBPR process. Microbes Environ 32: 260–267. 28890468 10.1264/jsme2.ME17020 PMC5606696

[B28] Okabe, S., Kamizono, A., Zhang, L., Kawasaki, S., Kobayashi, K., and Oshiki, M. (2024) Salinity tolerance and osmoadaptation strategies in four genera of anammox bacteria: Brocadia, Jettenia, Kuenenia, and Scalindua. Environ Sci Technol 58: 5357–5371. 38491939 10.1021/acs.est.3c07324

[B29] Okabe, S., Kamizono, A., Kawasaki, S., Kobayashi, K., and Oshiki, M. (2025) Interspecific competition and adaptation of anammox bacteria at different salinities: Experimental validation of the Monod growth model with salinity inhibition. Water Res 271: 122883. 39637692 10.1016/j.watres.2024.122883

[B30] Pinchuk, G.E., Ammons, C., Culley, D.E., Li, S.-M.W., McLean, J.S., Romine, M.F., et al. (2008) Utilization of DNA as a sole source of phosphorus, carbon, and energy by Shewanella spp.: ecological and physiological implications for dissimilatory metal reduction. Appl Environ Microbiol 74: 1198–1208. 18156329 10.1128/AEM.02026-07 PMC2258558

[B31] Planavsky, N.J., Asael, D., Rooney, A.D., Robbins, L.J., Gill, B.C., Dehler, C.M., et al. (2023) A sedimentary record of the evolution of the global marine phosphorus cycle. Geobiology 21: 168–174. 36471206 10.1111/gbi.12536

[B32] Ponce-Jahen, S.J., Cercado, B., Estrada-Arriaga, E.B., Rangel-Mendez, J.R., and Cervantes, F.J. (2024) Anammox with alternative electron acceptors: perspectives for nitrogen removal from wastewaters. Biodegradation 35: 47–70. 37436663 10.1007/s10532-023-10044-3 PMC10774155

[B33] Pynaert, K., Smets, B.F., Wyffels, S., Beheydt, D., Siciliano, S.D., and Verstraete, W. (2003) Characterization of an autotrophic nitrogen-removing biofilm from a highly loaded lab-scale rotating biological contactor. Appl Environ Microbiol 69: 3626–3635. 12788771 10.1128/AEM.69.6.3626-3635.2003 PMC161519

[B34] Quast, C., Pruesse, E., Yilmaz, P., Gerken, J., Schweer, T., Yarza, P., et al. (2012) The SILVA ribosomal RNA gene database project: improved data processing and web-based tools. Nucleic Acids Res 4: D590–D596. 10.1093/nar/gks1219 PMC3531112 23193283

[B35] Roques, J.A.C., Micolucci, F., Hosokawa, S., Sundell, K., and Kindaichi, T. (2021) Effects of recirculating aquaculture system wastewater on anammox performance and community structure. Processes 9: 1183.

[B36] Roques, J.A.C., Unegbu, E., Fujii, N., Marqué, A., Micolucci, F., Sundell, K.S., and Kindaichi, T. (2024) Tolerance of the marine anammox Candidatus Scalindua to high nitrate concentrations: implications for recirculating aquaculture systems. Water 16: 3705.

[B37] Sebastian, M., and Ammerman, J.W. (2009) The alkaline phosphatase PhoX is more widely distributed in marine bacteria than the classical PhoA. ISME J 3: 563–572. 19212430 10.1038/ismej.2009.10

[B38] Shoiful, A., Kambara, H., Cao, L.T.T., Matsushita, S., Kindaichi, T., Aoi, Y., et al. (2020) Mn(II) oxidation and manganese-oxide reduction on the decolorization of an azo dye. Int Biodeterior Biodegrad 146: 104820.

[B39] Si, P., Li, J., Xie, W., Dong, H., and Qiang, Z. (2021) Deciphering nitrogen removal mechanism through marine anammox bacteria treating nitrogen-laden saline wastewater under various phosphate doses: Microbial community shift and phosphate crystal. Bioresour Technol 325: 124707. 33482477 10.1016/j.biortech.2021.124707

[B40] Thingstad, T.F., Krom, M.D., Mantoura, R.F.C., Flaten, G.A.F., Groom, S., Herut, B., et al. (2005) Nature of phosphorus limitation in the ultraoligotrophic Eastern Mediterranean. Science 309: 1068–1071. 16099984 10.1126/science.1112632

[B41] Tom, A.P., Jayakumar, J.S., Biju, M., Somarajan, J., and Ibrahim, M.A. (2021) Aquaculture wastewater treatment technologies and their sustainability: A review. Energy Nexus 4: 100022.

[B42] Tomisaki, D., Kondo, T., Saito, Y., and Isaka, K. (2023) Effect of phosphorus limitation on the anammox process under different nitrogen concentrations. Biochem Eng J 200: 109092.

[B43] van de Graaf, A.A., de Bruijn, P., Robertson, L.A., Jetten, M.S.M., and Kuenen, J.G. (1996) Autotrophic growth of anaerobic ammonium-oxidizing micro-organisms in a fluidized bed reactor. Microbiology 142: 2187–2196. 10.1099/00221287-143-7-2415 33657728

[B44] van der Star, W.R.L., Abma, W.R., Blommers, D., Mulder, J.-W., Tokutomi, T., Strous, M., et al. (2007) Startup of reactors for anoxic ammonium oxidation: Experiences from the first full-scale anammox reactor in Rotterdam. Water Res 41: 4149–4163. 17583763 10.1016/j.watres.2007.03.044

[B45] Van Mooy, B.A.S., Fredricks, H.F., Pedler, B.E., Dyhrman, S.T., Karl, D.M., Koblížek, M., et al. (2009) Phytoplankton in the ocean use non-phosphorus lipids in response to phosphorus scarcity. Nature 458: 69–72. 19182781 10.1038/nature07659

[B46] Wanner, B.L. (1993) Gene regulation by phosphate in enteric bacteria. J Cell Biochem 51: 47–54. 8432742 10.1002/jcb.240510110

[B47] Yang, S., Xu, S., Florentino, A.P., Mohammed, A., Ashbolt, N.J., and Liu, Y. (2019) Importance of controlling phosphate concentration in nitritation–anammox reactor operation. Environ Sci: Water Res Technol 5: 1234–1243.

[B48] Yao, M., Elling, F.J., Jones, C., Nomosatryo, S., Long, C.P., Crowe, S.A., et al. (2016) Heterotrophic bacteria from an extremely phosphate‐poor lake have conditionally reduced phosphorus demand and utilize diverse sources of phosphorus. Environ Microbiol 18: 656–667. 26415900 10.1111/1462-2920.13063 PMC5872838

[B49] Zhang, Z.Z., Xu, J.-J., Hu, H.-Y., Shi, Z.-J., Ji, Z.-Q., Deng, R., et al. (2016) Insight into the short- and long-term effects of inorganic phosphate on anammox granule property. Bioresour Technol 208: 161–169. 26943933 10.1016/j.biortech.2016.02.097

[B50] Zhang, L., Narita, Y., Gao, L., Ali, M., Oshiki, M., and Okabe, S. (2017a) Maximum specific average growth rate of anammox bacteria revisited. Water Res 116: 296–303. 28347953 10.1016/j.watres.2017.03.027

[B51] Zhang, Z.Z., Hu, H.-Y., Xu, J.-J., Shi, Z.-J., Deng, R., Ji, Z.-Q., et al. (2017b) Effects of inorganic phosphate on a high-rate anammox system: Performance and microbial community. Ecol Eng 101: 201–210.

[B52] Zheng, L., Ren, M., Xie, E., Ding, A., Liu, Y., Deng, S., and Zhang, D. (2019) Roles of phosphorus sources in microbial community assembly for the removal of organic matters and ammonia in activated sludge. Front Microbiol 10: 1023. 31156575 10.3389/fmicb.2019.01023 PMC6532738

