# Supplementary material for: Optimal Phosphate Concentration for Growth and Normal Functioning of Marine Anammox Bacteria, Candidatus Scalindua sp
Source: Microbes Environ. 2025 Nov 12;40(4):ME25042. doi: 10.1264/jsme2.ME25042 (PMC12727191; doi:10.1264/jsme2.ME25042)
Supplement: Supplementary file 1 — Supplementary Material [file 40_25042_s1.pdf]

Supplementary Materials

Optimal Phosphate Concentration for Growth and Normal Functioning of Marine Anammox Bacteria, *Candidatus* Scalindua sp.

Thelwadanage Nadisha Tharangani Kumari Nawarathna, Haruhi Iida, Naoki Fujii, Noriatsu Ozaki, Akiyoshi Ohashi, Jonathan A.C. Roques, Tomonori Kindaichi

The supplementary material contains one supplemental method, 4 figures, and 1 table.

Supplemental Method

Calculation method of the total copy number in the effluent samples

The total copy numbers of effluent biomass samples shown in Figure 3 were calculated from the following procedures.

1. Gene copy number per L of effluent (copies/L-eff) for each interval (10-days or 20-day only for 40-60 days)

The number of gene copies per L of effluent was calculated using the following equation.

Gene copies per L of effluent =

$$\frac{\text{qPCR quantity}\left(\frac{\text{copies}}{\mu\text{L}-\text{DNA extract}}\right)\times\text{DNA extract volume }(500\ \mu\text{L}-\text{DNA extract / L}-\text{Eff})}{\text{Filtration volume (L}-\text{Eff)}}$$

2. Gene copy for 10 days of effluent

For Figure 3, gene copies per L of effluent were scaled to 10-day effluent volume.

Gene copy for 10 days of effluent = gene copies per L effluent (copies/L-eff) × flow rate of each reactor (4.32 L/day) × 10 days (or 20 days).

3. Average copies for 10 days (or 20 days)

For each sampling day, triplicate values of Gene copy for 10 days of effluent were obtained.

Copies= $\frac{X_1+X_2+X_3}{3}$

Where X_1, X_2, and X_3 are copies for Gene copy for 10 days of effluent of each replicate.

4. Total average copy numbers

In this study, we defined the Total copy numbers (Copies) as shown in Fig. 3 was calculated using the following equation.

Total copy numbers (Copies) = Average copies days 0-10 + Average copies days 10-20 + Average copies days 20-30 + Average copies 30-40 + Average copies days 40-60 + Average copies days 60-70

The errors were calculated based on the law of error propagation.

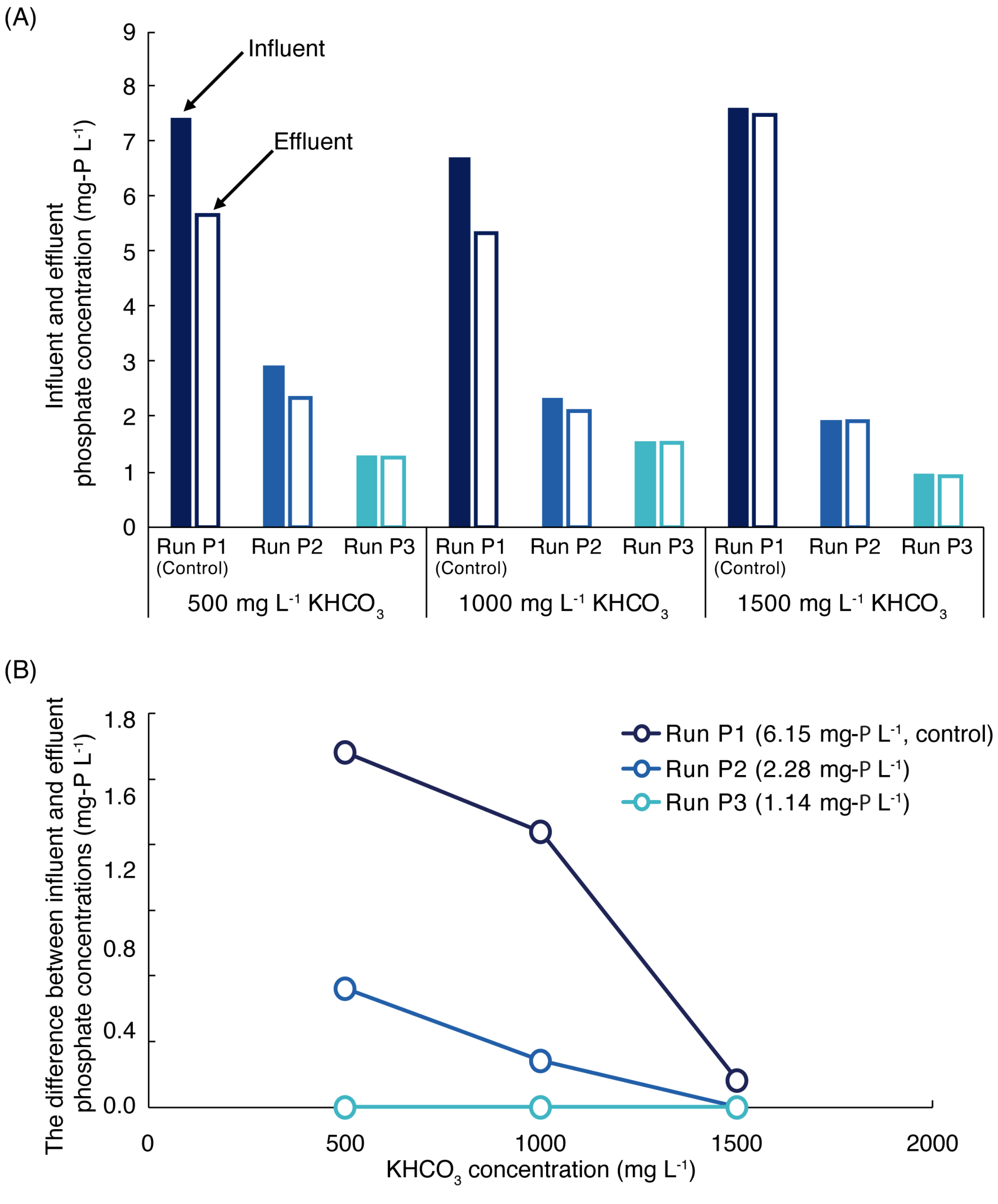

Figure S1

Optimizing phosphate removal with bicarbonate addition. (A) Influent and effluent phosphate concentrations under different bicarbonate conditions. (B) Relationship between influent bicarbonate and phosphate precipitation concentration.

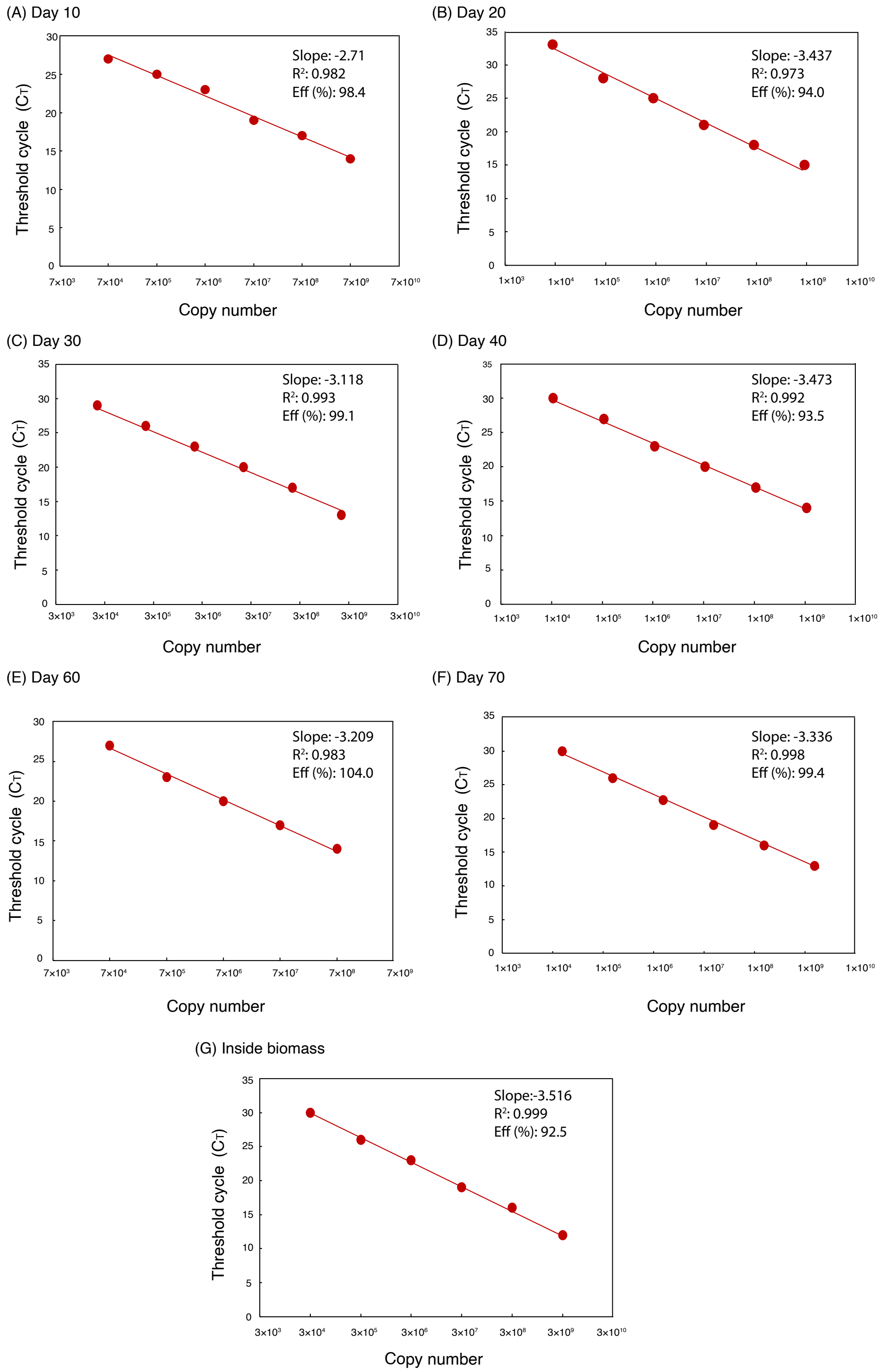

Figure S2

Standard curves of *Ca*. Scalindua qPCR for the measurement of effluent samples (A-F) and reactor biomass samples (G) using 10-fold serial dilutions of plasmid DNA carrying *Ca*. Scalindua 16S rRNA genes. The slope, coefficient of determination (R^2), and amplification efficiency are also shown in the figures.

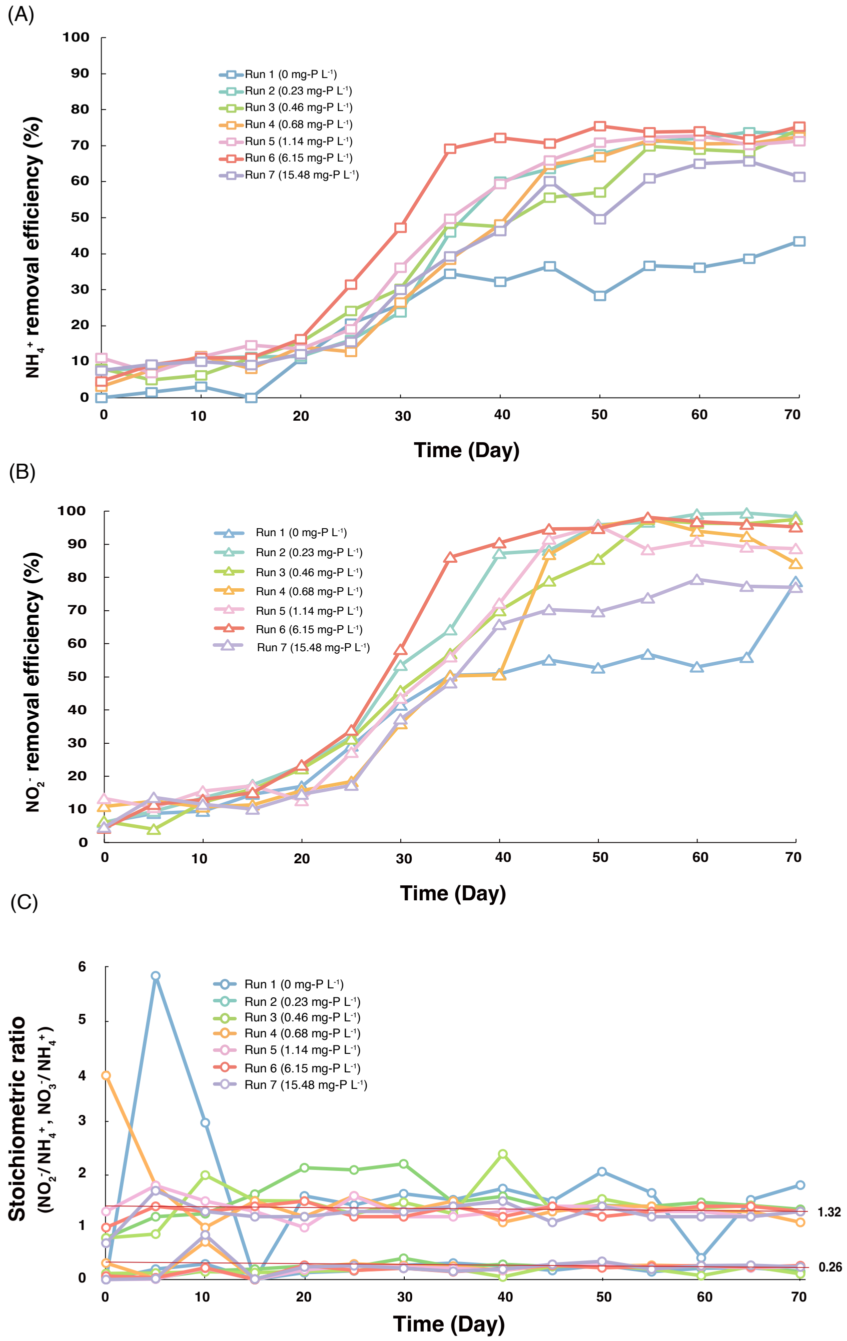

Figure S3

The NH_4^+ (A) and NO_2^- (B) removal efficiency, and anammox stoichiometric ratios (C) with different phosphate concentrations. The values of $\Delta NO_2^-/\Delta NH_4^+ = 1.32$ and $\Delta NO_3^-/\Delta NH_4^+ = 0.26$ are shown in panel (C).

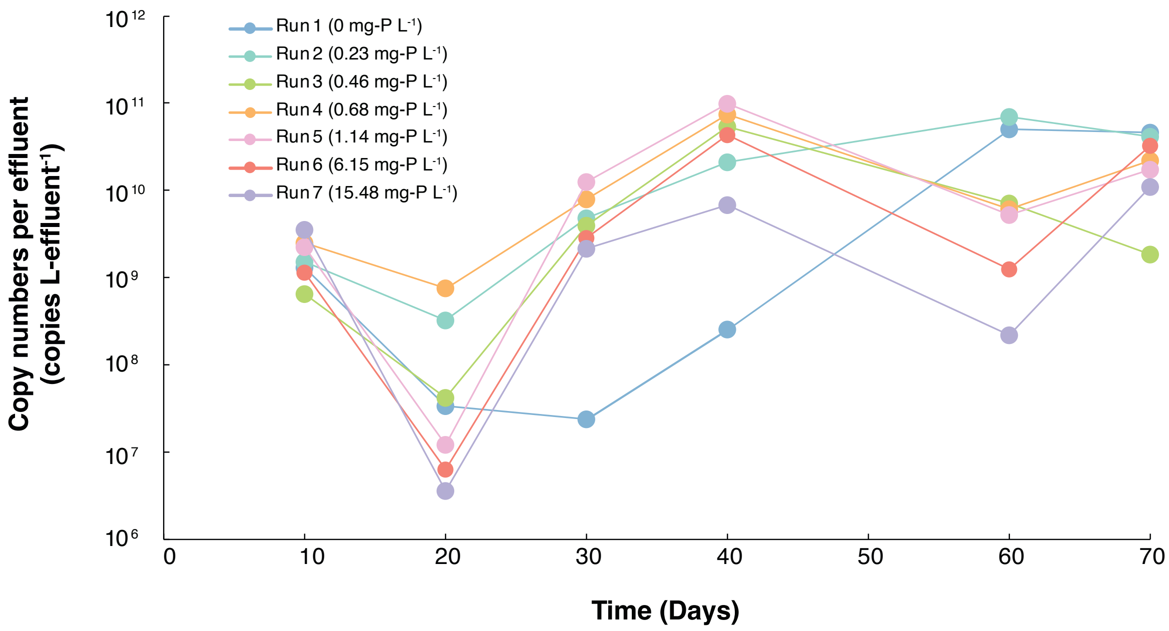

Figure S4

Copy numbers of the 16S rRNA gene of *Ca*. Scalindua in the effluent samples during the 70 days operation.

Table S1: Composition of synthetic wastewater under different phosphate and bicarbonate concentrations in the abiotic experiment.

Compounds	Unit	Run P1 (Control)	Run P2	Run P3
SEALIFE	[g L^{-1}]	35	35	35
$(NH_4)_2SO_4$	[mg-N L^{-1}]	31.8	31.8	31.8
$NaNO_2$	[mg-N L^{-1}]	30.4	30.4	30.4
$KHCO_3$	[mg L^{-1}]	500/1000/1500	500/1000/1500	500/1000/1500
$MgSO_4 \cdot 7H_2O$	[mg L^{-1}]	300	300	300
$CaCl_2 \cdot H_2O$	[mg L^{-1}]	180	180	180
KH_2PO_4	[mg-P L^{-1}]	6.15	2.28	1.14
TE I	[mL L^{-1}]	1	1	1
TE II	[mL L^{-1}]	1	1	1
